# FGF23, Hypophosphatemia, and Emerging Treatments

**DOI:** 10.1002/jbm4.10190

**Published:** 2019-05-13

**Authors:** Erik A Imel, Andrew Biggin, Aaron Schindeler, Craig F Munns

**Affiliations:** ^1^ Division of Endocrinology Indiana University School of Medicine, Indianapolis, IN USA; ^2^ The University of Sydney Children's Hospital Westmead Clinical School, University of Sydney Sydney Australia; ^3^ Department of Endocrinology The Children's Hospital at Westmead Westmead Australia; ^4^ Orthopaedic Research Unit, The Children's Hospital at Westmead Westmead Australia

**Keywords:** FGF23, KLOTHO, XLH, X‐LINKED HYPOPHOSPHATEMIA, BUROSUMAB

## Abstract

FGF23 is an important hormonal regulator of phosphate homeostasis. Together with its co‐receptor Klotho, it modulates phosphate reabsorption and both 1α‐hydroxylation and 24‐hydroxylation in the renal proximal tubules. The most common FGF23‐mediated hypophosphatemia is X‐linked hypophosphatemia (XLH), caused by mutations in the *PHEX* gene. FGF23‐mediated forms of hypophosphatemia are characterized by phosphaturia and low or low‐normal calcitriol concentrations, and unlike nutritional rickets, these cannot be cured with nutritional vitamin D supplementation. Autosomal dominant and autosomal recessive forms of FGF23‐mediated hypophosphatemias show a similar pathophysiology, despite a variety of different underlying genetic causes. An excess of FGF23 activity has also been associated with a number of other conditions causing hypophosphatemia, including tumor‐induced osteomalacia, fibrous dysplasia of the bone, and cutaneous skeletal hypophosphatemia syndrome. Historically phosphate supplementation and therapy using analogs of highly active vitamin D (eg, calcitriol, alfacalcidol, paricalcitol, eldecalcitol) have been used to manage conditions involving hypophosphatemia; however, recently a neutralizing antibody for FGF23 (burosumab) has emerged as a promising treatment agent for FGF23‐mediated disorders. This review discusses the progression of clinical trials for burosumab for the treatment of XLH and its recent availability for clinical use. Burosumab may have potential for treating other conditions associated with FGF23 overactivity, but these are not yet supported by trial data. © 2019 The Authors. *JBMR Plus* published by Wiley Periodicals, Inc. on behalf of American Society for Bone and Mineral Research.

## Introduction

Fibroblast growth factors (FGFs) are a diverse family of polypeptides involved with cell signaling that can have autocrine, paracrine, or hormonal activities.^1^ The majority of this expanding group of FGF ligands interact with one or more of the four signaling tyrosine kinase receptors (FGFR1‐4) at the cell surface, but a minority solely act as intracellular signaling molecules. FGFs are important for development and normal cell cycle regulation; altered FGF signaling underlies many congenital disorders and cancer subtypes.^2^ Preclinical models have been important in defining roles for FGFs and their associated receptors throughout embryonic development and postnatal life.^3^

FGF23 has emerged as a key regulatory factor for phosphate homeostasis and has been linked with a range of clinical conditions.^4^ FGF23 is produced primarily in bone, while its primary hormonal function is to regulate phosphate homeostasis and vitamin D metabolism. This review will outline the role of FGF23 as a regulator of circulating phosphate levels via pathways involving its co‐receptor Klotho, activated vitamin D, and parathyroid hormone (PTH); the role of FGF23 in heart and kidney disease, hypophosphatemic rickets, and other conditions associated with FGF23 dysregulation; and lastly emerging therapeutics for treating FGF23‐mediated hypophosphatemia.

## FGF23 Regulates Serum Phosphate

Phosphate is an inorganic molecule with a critical biological role that is regulated via a multifaceted homeostatic mechanism. The major organs that modulate phosphate levels are the digestive system, the kidneys, the bone, and the parathyroid gland,^5^ with a number of key molecular factors enabling cross‐talk between the organ systems. These include FGF23 and its co‐receptor Klotho, PTH, and 1,25‐dihydroxy vitamin D (1,25(OH)_2_D). Phosphate is taken up from food via the gut (20 mg/kg/d) and lost to the feces (7 mg/kg/d) and urine (13 mg/kg/d). Bone acts as a phosphate store, and normal bone turnover of phosphate is 3 mg/kg/d from both formation and resorption.^6^ The action of the kidneys is critical to maintaining homeostasis because loss in the proximal renal tubule can be restricted or increased by modifying reabsorption in response to phosphate imbalance via signals from PTH and FGF23.

Under normal physiological conditions, FGF23 is primarily expressed by the bone and functions as a global regulator of phosphate homeostasis. Unlike FGFs in other subfamilies, FGF23 has a low affinity for heparin sulfate, allowing it to enter the circulation and act as an endocrine hormone. Absence of dentin matrix protein 1 (DMP‐1) and phosphate‐regulating gene with homologies to endopeptidases on the X chromosome (PHEX) influences expression of FGF‐23 in bone and mutations in the encoding genes result in congenital hypophosphatemia.^7^

FGF23 binding to FGFRs requires a co‐receptor, Klotho, which acts as a scaffold for docking with FGFR1 (IIIc). Klotho (αKlotho) was originally described in a mouse model of premature aging but subsequently revealed to be a critical factor in modulating FGF23 activity in the kidneys.^8^, ^9^ In the kidneys, FGF23/Klotho function to suppress phosphate transporters in the proximal renal tubules, reducing the reabsorption of filtered phosphate.^10^, ^11^ Furthermore, FGF23 can inhibit expression of 1α‐hydroxylase while increasing expression of 24‐hydroxylase, thus reducing the concentration of active 1,25(OH)_2_D.^10^, ^11^ Several recent reviews comprehensively summarize the capacity of FGF23 and Klotho to facilitate this inter‐organ communication.^12^, ^13^

1,25(OH)_2_D is a critical regulator of calcium homeostasis and is converted from 25(OH)D in the kidney via 1α‐hydroxylase, which is encoded by CYP27B1. This conversion can be induced by the hormone PTH, while CYP27B1 expression is inhibited by FGF23 and by 1,25(OH)_2_D itself.^14^ 1,25(OH)_2_D production may be indirectly increased in the setting of hypocalcemia and hypophosphatemia or decreased in the setting of hypercalcemia and hyperphosphatemia, likely via corresponding changes in PTH and FGF23. 1,25(OH)_2_D acts to regulate calcium and phosphate, stimulating active absorption from the gut and modulating their release from bone. Administration of 1,25(OH)_2_D can also acutely increase FGF23 levels in mice^15^ and humans,^16^ providing an additional level of cross‐talk between the two systems.

PTH is a polypeptide hormone secreted by the parathyroid gland that has a substantive role in the regulation of calcium homeostasis and bone growth. PTH expression is primarily regulated by changes in serum calcium, though increases in phosphate intake may lead to indirect increases in PTH due to the interplay between serum calcium and phosphate levels. High systemic levels of PTH increase osteoclastic bone turnover, liberating calcium and phosphate stores from the bone. In preclinical models, recombinant FGF23 was found to increase parathyroid Klotho levels and activate MAPK signaling, leading to a suppression of PTH expression over short time courses.^17^ However, it is also noted that genetic models of hypophosphatemia due to chronic elevated FGF23, such as XLH, are associated with elevated PTH concentrations, even in the absence of phosphate treatment.^18^, ^19^ This mild secondary hyperparathyroidism may be attributable to FGF23 excess inducing a relative deficiency in 1,25(OH)_2_D.

## FGF23, Chronic Kidney Disease, and Cardiac Hypertrophy

Chronic kidney disease (CKD) is a major and growing health challenge and one that is associated with massive increases in circulating FGF23.^20^ There is evidence that this is mediated by Klotho expression in the bone compartment, supported by renal failure models in limb‐specific Klotho knockout mice.^21^ Dysregulated FGF23 signaling may also contribute to secondary hyperparathyroidism in CKD.^22^ Papers modeling the role of FGF23 in CKD have led to speculations that FGF23‐targeted therapeutic interventions may have beneficial effects in the context of kidney disease.^23^ However, in an animal model of CKD, blocking FGF23 with an antibody worsened their hyperphosphatemia and led to earlier death.^24^ This is likely due to blocking FGF23 without fixing the hyperphosphatemia of CKD, thus impairing an endogenous compensating mechanism. Because of this, kidney function also needs to be considered when treating hypophosphatemic disorders.

FGF23 is produced primarily by osteoblasts and osteocytes; however, it can be pathologically upregulated in other disease states such as hypertrophic cardiomyopathy.^4^ Indeed it has been long argued that the increases in FGF23 are causal for cardiac hypertrophy,^25^ although via Klotho‐independent processes. However, recent studies using mouse models of FGF23 knockout and upregulation do not support this supposition,^26^, ^27^ and individuals with primarily FGF23‐related hypophosphatemic bone disease do not consistently present with ventricular hypertrophy.^28^ As with CKD, the difference may be due to whether elevations of FGF23 are occurring during hyperphosphatemia or hypophosphatemia.

## FGF23 and Hypophosphatemia (Table 1)

**Table 1:** Disorders Associated With FGF23 and Abnormal Phosphate Regulation

Disorder	Gene	Phenotype
General features of all disorders listed below		Hypophosphatemia, renal phosphate wasting, elevated FGF23, low or normal 1,25(OH)_2_D, variable skeletal deformities due to rickets/osteomalacia, pseudofractures
X‐linked hypophosphatemia (XLH)	*PHEX*	Dental abscesses, enthesopathy (X‐linked dominant)
Autosomal dominant hypophosphatemic rickets (AD)	*FGF23*	Dental abscesses, enthesopathy, waxing and waning of clinical phenotype with variable age of onset, association of elevated FGF23 with iron deficiency
Autosomal recessive hypophosphatemic rickets (AR)	*DMP1*	Dental abscesses, enthesopathy
	*ENPP1*	Can have generalized arterial calcification beginning in infancy
	*FAM20C*	Severe dental disease, intracerebral calcifications, osteosclerosis, additional dysmorphic facies
Fibrous dysplasia	*GNAS*	One or more skeletal lesions of fibrous dysplasia, may have associated nerve compression, localized skeletal fragility, and may be present as part of McCune‐Albright syndrome with café au lait macules and various hormone hypersecretion (resulting in precocious puberty, acromegaly, hyperthyroidism or other hormone excesses)
Cutaneous skeletal hypophosphatemia syndrome (CSHS)	*RAS (HRAS, NRAS)*	Large epidermal nevi or congenital melanocytic nevi, dysplastic skeletal lesions
Tumor‐induced osteomalacia (TIO)		Acquired condition with about half having translocations resulting in fusion proteins FN1‐FGFR1 or FN1‐FGF1; tumors in any location, which may be small and difficult to localize

### X‐linked hypophosphatemia (XLH)

XLH is a rare congenital bone condition caused by inactivating mutations in the *PHEX* gene, which leads to upregulation of FGF23 from the bone compartment and resultant hypophosphatemia.^29^ Sporadic cases appear to represent about 20% to 30% of cases.^30^ XLH is often mistaken for the more common nutritional rickets, with children with XLH showing increased serum alkaline phosphatase activity as well as lower‐extremity bowing, rachitic features, and/or metaphyseal dysplasia. However, the condition is nonresponsive to nutritional vitamin D treatment because it is a consequence of renal phosphate wasting along with impaired activation of vitamin D, both induced by elevations of FGF23.^31^ Failure of clinical laboratories to use the higher age‐appropriate normal ranges of phosphate in children is still common and often leads to delayed diagnosis as well.

**Figure 1:**
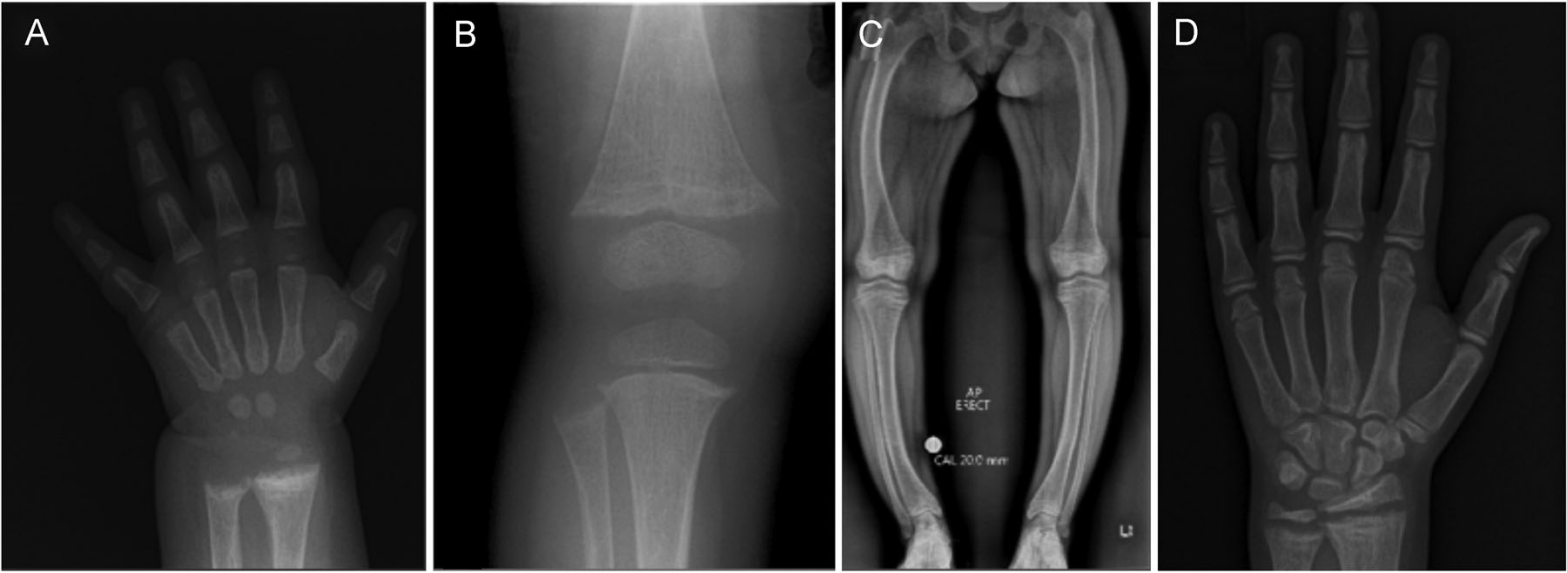
A now 14‐year‐old female with XLH (PHEX:c.[151C>T];[=] p.[Gln51*]) diagnosed at 7 months of age. (*A*) Radiograph of left hand and wrist at diagnosis with rachitic changes of distal radius and ulna and lacey appearance of bone. (*B*) Radiograph of right knee at 18 months old while treated with phosphate and calcitriol. There is fraying and splaying at the metaphyses and early cupping noted at the distal femur as well as the proximal tibia and fibula. (*C*) At 14 years old, she was managed with phosphate and calcitriol. She had short stature, normal ALP, and mild elevation in PTH. Symptoms included persistent ankle pain and waddling gait. There was also lateral bowing of both femora and tibias with widening of the proximal tibial growth plate. (*D*) Left hand radiograph at age 14 years showing widening of the proximal radius and ulna growth plates with evidence of rachitic changes.

Patients with XLH are not short at birth, and evidence of rickets is not immediately present.^32^, ^33^ Early diagnosis is useful and is most likely to occur in children of affected parents. On rare occasion, even using age‐appropriate normal ranges, we have observed false‐positive or false‐negative results when testing serum phosphate during the first few months after birth, requiring confirmation with repeat testing. Bowing deformities of legs usually develop after weight bearing begins, and around this time, growth impairments also become evident.^32^, ^33^ Rachitic features include bowing of long bones, genu varum, or valgum, along with abnormalities of the skull shape including frontal bossing, dolicocephaly, and flattening of the cranial base (Fig. 1). Craniosynostosis and Chiari malformations may occur.^31^, ^34^, ^35^ During growth, the leg length is disproportionately affected compared with the trunk length, and despite treatment, patients fail to have catchup growth during puberty, actually decreasing height *Z*‐scores during this time and are often short as adults.^36^

Additional complications of XLH include enthesopathy, osteoarthritis, pseudofractures, and dental abscesses. Severe dental disease is present in more than half of patients with XLH and is associated with abnormally mineralized dentin and cementum.^37^, ^38^, ^39^, ^40^, ^41^ Enthesopathy and osteoarthritis may severely limit range of motion and contribute to pain in patients with XLH.^42^, ^43^ The occurrence of spinal stenosis and its consequences in these patients is an under‐recognized issue, impairing quality of life. Patients may also have bone pain from osteomalacia itself, along with poorly healing chronic pseudofractures in up to half of adults.^43^ Muscle weakness is also present to some degree in many patients.^44^

Recently, the anti‐FGF23 antibody burosumab has emerged as a novel and transformational treatment strategy for XLH that targets the chronic upregulation of FGF23. Burosumab is expanded upon subsequently.

### Autosomal‐recessive and autosomal‐dominant hypophosphatemic rickets (ARHR, ADHR)

ARHR and ADHR are rare genetic bone disorders that also feature hypophosphatemia. ARHR has been linked to inactivating mutations in the *DMP1*, *FAM20C*, and *ENPP1* genes.^45^ Patients with DMP1 mutations are phenotypically similar to XLH. *ENPP1* mutations are associated with a generally severe phenotype of generalized arterial calcification of infancy; however, some patients may present with hypophosphatemia alone and its skeletal consequences in the absence of apparent arterial calcification.^46^, ^47^ *FAM20C* mutations have been reported in Raine syndrome, though some have hypophosphatemia.^48^ Patients with FAM20C may have severe dental disease, intracerebral calcifications, and osteosclerosis of long bones.

ADHR is linked to mutations in *FGF23* that stabilize the protein product, leading to increased FGF23 activity.^31^ Recent data indicate that patients with ADHR do not always express elevated levels of FGF23 or hypophosphatemia. In fact, some patients never manifest the disease (incomplete penetrance), while some affected patients spontaneously normalize. In the setting of iron deficiency, FGF23 gene expression increases.^49^ The normal FGF23 protein is able to be cleaved readily to maintain normal intact FGF23 levels even when iron deficient. However, the ADHR mutation creates an FGF23 protein that resists cleavage.^14^ Thus, when iron deficiency drives an increase in FGF23 gene expression, the mutant FGF23 builds up, causing hypophosphatemia, while normalization of iron in ADHR has been associated with the normalization of the biochemical and skeletal phenotype.^50^ However, due to the potential for certain forms of intravenous iron to also precipitate acute increases in intact FGF23,^51^ we would avoid treating these iron‐deficient ADHR subjects with intravenous iron (especially iron polymaltose and iron carboxymaltose), as these could make the ADHR patient severely ill with profound hypophosphatemia. Thus we would recommend the oral route.

Like XLH, both ADHR and ARHR are clinically managed by dietary phosphate supplementation combined with calcitriol or other active vitamin D analogs.^52^ However, for ADHR, iron supplementation to normalize iron stores may be a safer and more appropriate therapy. The concept of treating ADHR with iron alone is supported by studies indicating that iron deficiency appears to be a key pathologic mechanism in ADHR^49^, ^50^; however, neither study tested the hypothesis of treating with iron. One case report described loss of the hypophosphatemic phenotype in an ADHR patient during iron supplementation.^53^ Preliminary data from a pilot clinical trial of oral iron therapy for ADHR indicated normalization of FGF23 and phosphate levels.^54^ Although this trial is not yet complete, subjects who had previously required calcitriol and phosphate were able to discontinue these as iron normalized.

## Conditions of Localized Increased FGF23 Production

Tumor‐induced osteomalacia (TIO) is caused by mesenchymal tumors producing an excess of FGF23. TIO is a rare paraneoplastic disorder causing complex symptomology that can feature chronic pain and/or muscle weakness with a postural deformity.^55^ Adult patients often present with osteomalacia and increased fracture incidence, whereas children are more likely to present with short stature and abnormal gait. Reduced serum 1,25(OH)_2_D and elevated bone‐specific alkaline phosphatase can be used as diagnostic markers, and if measured, serum FGF23 is typically elevated.^56^ In a large TIO series, 48% of tumors exhibited evidence for translocations resulting in abnormal fusion proteins of fibronectin 1 (FN1) and either fibroblast growth factor receptor 1 (FGFR1) or fibroblast growth factor 1 (FGF1) (3/50 [6%] FN1‐FGFR1 and 21/50 [42%] FN1‐FGF1), hypothesized to generate an autocrine function loop.^57^ Complete removal of the tumor by wide resection or ablation is typically curative of the biochemical abnormalities with improvement in bone and muscle strength.^58^ However, these mesenchymal tumors are slow growing and may be difficult to localize. Patients often require medical therapy with vitamin D analogs and phosphate. However, FGF23‐targeted therapies may be applicable for managing the hypophosphatemic osteomalacia.^59^

Fibrous dysplasia (FD) occurs after postzygotic mutations in *GNAS* and can result in elevated FGF23 secretion by the FD tissue.^60^ This is particularly pertinent to patients with McCune‐Albright syndrome who can also have other associated endocrinopathies.^61^ FD can be localized to a single area (monostotic) or be part of a more generalized pattern (polyostotic or panostotic) affecting the craniofacial, axial, or appendicular skeleton. The number and size of FD lesions increases with age during childhood, but after age 15 years, few new lesions present.^62^ Children usually present with bone pain, deformity, or pathological fracture. Typical radiograph features include a characteristic “ground glass” appearance.^63^ The development of elevated FGF23 and of associated hypophosphatemia correlates with the skeletal burden of FD.^64^ Patients with hypophosphatemia are managed with active vitamin D and phosphate similar to other FGF23‐mediated hypophosphatemia. Bisphosphonates have been used to manage the bone pain associated with FD but do not improve disease progression.^65^

Cutaneous skeletal hypophosphatemia syndrome (CSHS), also called epidermal nevus syndrome or linear sebaceous nevus syndrome in various reports, presents with epidermal nevi, or less commonly congenital melanocytic nevi of variable sizes, along with hypophosphatemia and dysplastic bone lesions.^66^ CSHS is caused by somatic mutations in RAS (HRAS, NRAS) present in both the skin lesions and the dysplastic bone lesions.^67^ Elevated plasma FGF23 concentrations cause the hypophosphatemia and resultant osteomalacia.^67^ Although the skin lesions were proposed as the source of FGF23 and some studies suggested improvement in hypophosphatemia after resection of the nevus,^68^ subsequent studies have not demonstrated convincing improvement after resection of these generally large skin lesions.^66^ In contrast, the FGF23 expression was not detectable in the skin lesions.^67^ Because the RAS mutations are detectable within the dysplastic bone lesions, which are somewhat similar to those in fibrous dysplasia, and bone is the usual source for FGF23, these lesions are thought to be the source of excess FGF23 production. Skeletal deformity from dysplasia, rickets, leg bowing, fractures, and scoliosis are not uncommon. Phosphate and active vitamin D analogs have been shown to be effective for patient treatment.^66^

## Medical Therapy of FGF23‐Mediated Skeletal Disorders

Although this review will concentrate on medial therapy, the importance of a multidisciplinary team approach to the management of children and adults with FGF23‐related bone disease should not be overlooked. In the opinion of the authors, a coordinated and patient‐centered management plan offers the best possibility for optimal outcome. Throughout life, the important treatment goals of improved quality of life, attainment of developmental milestones, reduction in pain, maximization of mobility, and inclusion in society should be sought. To attain these, the health care team is likely to include professionals from physiotherapy, occupational therapy, genetic counseling, orthopedic surgery, pediatrics/medicine (endocrinology, nephrology, general practice), social work, and dental providers.

Historically the treatment of FGF23‐mediated skeletal disorders has involved supplementation with 1‐hydroxylated vitamin D analogs and phosphate. The goal with this therapy is not specifically to normalize serum phosphate, and one should never treat XLH with phosphate alone as active vitamin D analogs are necessary for effective treatment and to combat the tendency of phosphate dosing to cause hyperparathyroidism. Although this treatment does improve the osteomalacia and rickets, outcomes are highly variable, with many patients retaining significant skeletal deformity and short stature (Fig. 1).^31^ As a result, corrective surgeries are often required to manage skeletal deformity and pain and improve quality of life. Furthermore, there is no evidence of benefit of any treatment modality on enthesopathy. Adult XLH patients often require surgical intervention for pseudofractures, spinal stenosis, or joint replacements. Dental interventions are also frequently necessary for abscessed teeth and periodontitis. Retrospective studies suggest that treatment with phosphate and active vitamin D analogs reduce dental complications in XLH.^40^, ^41^

Therapy with calcitriol and phosphate salts is often limited by gastrointestinal side effects, as well as by more serious complications, including development of hypercalcemic hyperparathyroidism and nephrocalcinosis.^69^ Furthermore, the hyperparathyroidism involves multigland disease, which may be difficult to manage even with surgery. Limited data suggest that cinacalcet, a calcimimetic, may be able to ameliorate the hyperparathyroidism in some patients with XLH.^70^, ^71^, ^72^ Phosphate dosing causes temporary rises of PTH, but single doses of cinacalcet to children with XLH without hyperparathyroidism blocked the rise in PTH after a subsequent phosphate dose.^72^ Furthermore, PTH stimulates FGF23 secretion from osteocytes.^73^ So modulating PTH might have some effects to decrease FGF23 or at the very least could decrease the PTH effect on the proximal renal tubule to promote phosphaturia. Two TIO patients who were treated with cinacalcet demonstrated improvements in tubular reabsorption of phosphate and serum phosphate.^74^ In a case report, addition of cinacalcet to a child refusing phosphate showed improvement in rickets.^75^ These data collectively suggest potential for a calcimimetic to be used for XLH and to prevent the development of hyperparathyroidism in patients as well as to treat hyperparathyroidism. However, overall data in XLH are limited and data are generally lacking on long‐term administration of cinacalcet to nonhyperparathyroid XLH patients.

Nephrocalcinosis complicates 50% to 80% of treated patients, and its influence on CKD in the long term remains uncertain. One study reported normal renal function 25 years after acute vitamin D toxicity with nephrocalcinosis in two XLH patients.^76^ However, other authors found that 9.1% of adults with XLH had CKD.^77^ The authors have also managed XLH patients with CKD stages 3 to 5 as adults. Hypertension is also common in these patients and may relate to lower glomerular filtration rates.^77^ In general many of these complications may be the result of failing to adequately balance the risks and the benefits when manipulating therapeutic doses.

Growth hormone has been used to treat short stature in children with XLH. However, despite apparent improved linear growth over 3 years in a randomized open‐label study of XLH patients,^78^ there was no significant difference in final adult height between those treated with growth hormone and controls.^79^ Interestingly, the randomized study excluded patients with severe leg deformities, meaning that we have no controlled trial data on the potential for benefit or harm in that group. Consequently, growth hormone use should still be considered experimental in XLH.

Calcitonin has been studied in humans with XLH. Calcitonin increases 1α‐hydroxylase expression in the XLH mouse model and 1,25(OH)_2_D levels in humans with XLH after a single injection.^80^, ^81^, ^82^ However, calcitonin also decreased FGF23 in patients with XLH after a single injection.^80^ A three‐month trial of calcitonin nasal spray did not improve serum FGF23 or phosphate, lowering enthusiasm for this as an alternate therapy.^83^

Additional potential strategies to manage FGF23 excess include trying to decrease FGF23 production or blocking FGF23 activity at its receptor. Most of these strategies have only been studied in mouse models. Hexa‐D‐arginine is a small molecule that increases the expression of a helper protein 7B2, which enhances the activity of a subtilisin‐like proprotein convertase 2 (SPC2). In cell culture and the *hyp* (XLH) mouse model, hexa‐D‐arginine acting through this enzyme and downstream pathways was able to decrease FGF23 mRNA expression and protein concentration, with beneficial effects on the biochemical and skeletal phenotype.^84^ Other investigators have evaluated methods to block the FGFR using C‐terminal FGF23 fragments as an antagonist. Repeated injection of these fragments was able to increase renal sodium phosphate transporters in *hyp* mice, improving phosphate excretion, serum phosphate, and skeletal features.^84^ However, the treatment did not alter 1,25(OH)2D levels. FGFR blockade may also be accomplished through using small molecule FGFR inhibitors, such as those being studied as cancer therapies. Using a pan‐specific FGFR inhibitor, Wohrle and colleagues tested this strategy in the *hyp* mice.^85^ The FGFR inhibitor increased serum phosphate and 1,25(OH)2D in these mice, though phosphate remained low. The *Hyp* mice also improved longitudinal growth and decreased unmineralized osteoid. As this drug strategy is already being studied for human cancers, it also has potential for human application in FGF23 excess disorders including TIO.

## Therapeutic Potential of Burosumab, a Neutralizing Anti‐FGF23 Human Monoclonal Antibody

Burosumab (previously called KRN23) is a recombinant human IgG1 monoclonal antibody targeted to FGF23 that was developed for the treatment of XLH. The narrative for the development of burosumab is one of systematic drug development and validation. After the identification of FGF23/Klotho and a regulator of hypophosphatemia, this led to the development of antibodies to neutralize the action of FGF23.^86^ The *Hyp* mouse model features a 10‐ to 20‐fold increase in circulating FGF23 and develops hypophosphatemia, and this phenotype was improved by neutralizing FGF23 antibodies.^87^

Several clinical trials describe the effects of burosumab in children and adults with XLH. The first published trial was a randomized controlled trial of 38 adult XLH patients who received burosumab (or placebo) as a single i.v. or s.c. dose as part of an immunogenicity, safety, and tolerability trial.^88^ Positive effects were observed on serum phosphate, and while nausea, dizziness, dysgeusia, headache, and blood‐pressure increases were reported, no serious adverse events including hypercalciuria or hypercalcemia were observed. As a result of this trial, development progressed using a s.c. dosing regimen. This was followed by an open‐label phase 1/2 multi‐dose, dose finding study and its extension, showing that 4 weekly (Q4W) burosumab administration increased serum phosphate, 1,25(OH)_2_D, and renal tubular maximum reabsorption rate of phosphate relative to glomerular filtration rate (TmP/GFR) in adults with XLH with a peak and trough pharmacodynamic effect in these three variables supporting every 4‐week dosing.^89^ Analysis of the pharmacokinetic and pharmacodynamic profile of burosumab in adult XLH cohorts receiving Q4W dosing indicated maximal serum concentrations 7.0 to 8.5 days after dosing and a mean half‐life of 16.4 days.^90^, ^91^

Notably, an effect found in these initial trials and in all subsequent published burosumab trials in XLH to date was that a very high peak in serum 1,25(OH)_2_D occurs approximately 3 to 7 days after the first few s.c. doses.^89^, ^92^ Similar peak and trough effects occur during repeated dosing. However, with long‐term repeated dosing, the magnitude of that peak in 1,25(OH)_2_D lessens, while the peak effect on phosphate generally persists.^43^, ^89^, ^92^ This 1,25(OH)_2_D effect suggests that vitamin D activation is being suppressed in the pretreatment state by high FGF23 concentrations but that the cellular machinery is in place to support a rapid switch to producing 1,25(OH)_2_D. This is consistent with studies in the XLH mouse model indicating that despite high FGF23, the gene expression for the enzyme 1‐alpha hydroxylase is actually increased compared with normal mice, but the expression of the protein itself and its enzyme activity are lower than normal due to posttranscriptional or posttranslational effects.^93^ In that setting, acute removal of FGF23 effect (by burosumab, for example) would enable a rapid and intense response of 1,25(OH)_2_D. Thus, we speculate that after a prolonged period of improved phosphate metabolism, the background need for 1,25(OH)_2_D is lessened, resulting in lower peaks of 1,25(OH)_2_D after burosumab dosing. This concept is further supported by studies showing that the acute reductions in FGF23 that follow tumor resection in TIO correlate with a rapid rise in 1,25(OH)_2_D to above‐normal levels followed by normalization.^94^ Likewise, other strategies to block FGF23 action, including ERK inhibition and pan‐FGFR inhibition, also acutely raise 1,25(OH)_2_D levels above normal in hypophosphatemic (*hyp*) mice followed by normalization.^95^, ^96^

During this burosumab trial, improvements were also demonstrated in patient‐reported outcomes including physical functioning and stiffness scores using the Western Ontario and McMaster Osteoarthritis Index (WOMAC) scale.^97^ These improvements were generally supported by improvements in patient‐reported outcomes and functional outcomes in subsequent trials in children and adults.^43^, ^92^

Two key trials were published in 2018. A phase 2 randomized open‐label trial in 52 children aged 5 to 12 years compared second weekly (Q2W) and Q4W dosing in subjects previously treated with phosphate and active vitamin D analogs.^92^ All the children stopped phosphate and vitamin D to receive burosumab. Doses of burosumab were titrated to target serum phosphorus levels between 3.2 and 6.1 mg/dL (1.03 and 1.97 mmol/L). This trial demonstrated similar improvements in biochemical parameters as in the adult phase 2 trial. However, serum phosphorus maintained better values in the Q2W dosing group. Rickets improved in both groups as assessed using two different standardized tools to assess radiographic changes, with a suggestion of greater improvement in the Q2W group, and in the subgroup of patients having higher baseline rickets severity. Radiographic improvements were accompanied by improvements in alkaline phosphatase and small improvements in height *Z*‐score (+0.15). This trial lacked an active comparator or a placebo group but did suggest benefit from switching from conventional therapy to burosumab in these growing children. Similar findings of improvements in biochemical parameters, rickets, and leg deformities were recently published from an open‐label single‐arm 64‐week trial in 1‐ to 4‐year‐old children with XLH receiving Q2W burosumab.^98^ These children demonstrated increases in serum phosphorus and decreased rickets severity scores to a similar degree as in the older children.

Data from a 24‐week phase 3 randomized, double‐blind, placebo‐controlled burosumab trial were recently published.^43^ This study recruited 134 XLH adult patients into a multicenter trial with dosing of burosumab (*n* = 68) or placebo (*n* = 64) Q4W (1.0 mg/kg rounded to the nearest 10 mg, max 90 mg). As a whole, these patients had chronic pain per entry criteria, while roughly half had pseudofractures. Burosumab yielded significant and sustained improvements in serum phosphate and TmP/GFR. Transient increases in 1,25(OH)_2_D levels occurred chiefly 2 to 4 days after treatment. Over 24 weeks, significant improvements were found on the WOMAC physical function and stiffness scales, with the BPI Worst Pain measure showing a trend toward improvement that did not reach significance. Notably, a key secondary outcome of healing of active fractures and pseudofractures demonstrated differences between groups, with healing occurring by 24 weeks in 43.1% of the burosumab group and only 7.7% of placebo group with fractures. Two participants had serious adverse events, although these were not considered to be related to the study treatment. No discontinuations or dose‐limiting toxicities occurred. Of note, injection site reactions were of a lower frequency in the adults (11.8%) than in the pediatric phase 2 trial (57.7%), while the placebo‐treated adults had similar rates of injection‐site reactions (12.1%) to the burosumab‐treated adults. Interestingly restless legs syndrome was described in 17.9% of adults in the first multidose trial,^89^ though the mechanism is uncertain. In the randomized phase 3 trial, restless legs syndrome occurred in 11.8% of burosumab‐treated subjects but still occurred in 7.6% of placebo subjects, suggesting this may be an unrecognized complication of XLH.^43^

In April 2018, burosumab received FDA approval for clinical use for the treatment of XLH in adult and pediatric patients of 1 year of age and older. Based on the published trials, the recommended doses are 0.8 mg/kg every 2 weeks in children and 1 mg/kg every 4 weeks in adults. However, careful monitoring may identify patients in need of either dose increases or decreases. Some subjects in adult clinical trials have required dose decreases because of hyperphosphatemia. Patients should not take phosphate or active vitamin D analogs during burosumab treatment. Burosumab is not recommended in the setting of moderate to severe chronic kidney disease. Monitoring for nephrocalcinosis should still be conducted, as there is to date no evidence to tell if the long‐term risk of nephrocalcinosis is different from conventional therapy.

Additional questions remain. It remains to be determined for which patients the clinical outcome would be sufficient with conventional therapy and which adults or children would benefit the most from burosumab. Although the skeletal response to conventional therapy is noted to be quite variable, many patients do well with conventional therapy. Patients in clinical trials required a certain degree of severity at entry to be able to reliably measure and capture changes in outcomes. Given the high cost of burosumab compared with conventional therapy, the cost‐benefit ratio needs to be established, especially for those with milder disease, including potential differences in long‐term safety, which are largely unknown at the current time. Similarly, results of a phase 3 active comparator trial in children have not yet been published but may inform some of these questions. There is not any information to clarify what approach might be necessary in adolescents transitioning to adulthood. Likewise, there is no data on the safety of burosumab during pregnancy or lactation. It remains to be seen what the long‐term effect, if any, would be from prolonged burosumab treatment on achieved height at the end of growth, craniosynostosis in children, or on adult complications such as enthesopathy or osteoarthritis. Further, the effect of burosumab on dental health in XLH is yet to be explored and may not be adequately assessed in the short‐term clinical trials. However, because enthesopathy, arthritis, and dental disease exemplify some of the most debilitating features of the XLH, it is important to identify whether there could be positive (or negative) effects that could change the disease course. Further, the utility of burosumab for FGF23‐related skeletal disorders other than XLH is yet to be explored but may prove beneficial.

## Conclusions

Homeostatic regulation of serum phosphate levels involves coordination between multiple organ systems, with FGF23 and its co‐receptor Klotho being key regulatory factors. Disruption of these pathways causes systemic diseases with lifelong consequences that are difficult to manage, as exemplified by XLH. Anti‐FGF23 neutralizing antibody therapy such as burosumab has emerged as a safe and effective treatment for X‐linked hypophosphatemia and can rapidly stabilize serum phosphate levels and lead to improvements in rickets, skeletal healing, and physical function. Future basic and clinical research studies will need to address the utility of burosumab in treating other conditions associated with phosphate dysregulation, including other autosomal forms of hypophosphatemic rickets, and conditions such as TIO, FD, and CSHS.

## Disclosures

EAI and CM have research funding from Ultragenyx Pharmaceuticals. CM is a consultant for Kyowa Hakko Kirin Co., Ltd. All other authors state that they have no conflicts of interest.
